# Clinical Significance of Micronutrient Supplementation in Critically Ill COVID-19 Patients with Severe ARDS

**DOI:** 10.3390/nu13062113

**Published:** 2021-06-20

**Authors:** Quirin Notz, Johannes Herrmann, Tobias Schlesinger, Philipp Helmer, Stephan Sudowe, Qian Sun, Julian Hackler, Daniel Roeder, Christopher Lotz, Patrick Meybohm, Peter Kranke, Lutz Schomburg, Christian Stoppe

**Affiliations:** 1 Department of Anesthesiology, Intensive Care, Emergency and Pain Medicine, University Hospital Wuerzburg, D-97080 Wuerzburg, Germany; Herrmann_J4@ukw.de (J.H.); schlesinge_t@ukw.de (T.S.); helmer_p@ukw.de (P.H.); Roeder_d@ukw.de (D.R.); Lotz_C@ukw.de (C.L.); Meybohm_P@ukw.de (P.M.); Kranke_P@ukw.de (P.K.); christian.stoppe@gmail.com (C.S.); 2 Ganzimmun Diagnostics AG, D-55128 Mainz, Germany; dr.sudowe@ganzimmun.de; 3 Institute of Experimental Endocrinology, Charité-Universitätsmedizin Berlin, D-10115 Berlin, Germany; qian.sun@charite.de (Q.S.); julian.hackler@charite.de (J.H.); lutz.schomburg@charite.de (L.S.)

**Keywords:** acute respiratory distress syndrome, selen, zinc, critical care, oxidative stress, nutrient supplementation

## Abstract

The interplay between inflammation and oxidative stress is a vicious circle, potentially resulting in organ damage. Essential micronutrients such as selenium (Se) and zinc (Zn) support anti-oxidative defense systems and are commonly depleted in severe disease. This single-center retrospective study investigated micronutrient levels under Se and Zn supplementation in critically ill patients with COVID-19 induced acute respiratory distress syndrome (ARDS) and explored potential relationships with immunological and clinical parameters. According to intensive care unit (ICU) standard operating procedures, patients received 1.0 mg of intravenous Se daily on top of artificial nutrition, which contained various amounts of Se and Zn. Micronutrients, inflammatory cytokines, lymphocyte subsets and clinical data were extracted from the patient data management system on admission and after 10 to 14 days of treatment. Forty-six patients were screened for eligibility and 22 patients were included in the study. Twenty-one patients (95%) suffered from severe ARDS and 14 patients (64%) survived to ICU discharge. On admission, the majority of patients had low Se status biomarkers and Zn levels, along with elevated inflammatory parameters. Se supplementation significantly elevated Se (*p* = 0.027) and selenoprotein P levels (SELENOP; *p* = 0.016) to normal range. Accordingly, glutathione peroxidase 3 (GPx3) activity increased over time (*p* = 0.021). Se biomarkers, most notably SELENOP, were inversely correlated with CRP (r_s_ = −0.495), PCT (r_s_ = −0.413), IL-6 (r_s_ = −0.429), IL-1β (r_s_ = −0.440) and IL-10 (r_s_ = −0.461). Positive associations were found for CD8^+^ T cells (r_s_ = 0.636), NK cells (r_s_ = 0.772), total IgG (r_s_ = 0.493) and PaO_2_/FiO_2_ ratios (r_s_ = 0.504). In addition, survivors tended to have higher Se levels after 10 to 14 days compared to non-survivors (*p* = 0.075). Sufficient Se and Zn levels may potentially be of clinical significance for an adequate immune response in critically ill patients with severe COVID-19 ARDS.

## 1. Introduction

Since late 2019, the novel severe acute respiratory syndrome coronavirus 2 (SARS-CoV-2) has caused a dramatic outbreak of unusual viral pneumonia [1,2]. Being highly transmissible and infectious, coronavirus disease 2019 (COVID-19) has spread all over the world, posing significant threats to global public health. Among other symptoms, COVID-19 is characterized by fever and pneumonia, which in severe cases may lead to septic shock, hypoxemic respiratory failure, and acute respiratory distress syndrome (ARDS) [3]. These conditions are accompanied by distinct immunopathological changes such as lymphocytopenia and profuse inflammation, requiring prolonged and complex intensive care treatment [4]. Still, the pathogenesis and host response mechanisms in the progress of severe COVID-19 are only partially understood. However, increasing evidence suggests that excessive levels of reactive oxygen species (ROS), resulting from dysfunctional energy production on the mitochondrial level, perpetuate a vicious cycle between inflammation and oxidative stress [5]. Tumor necrosis factor (TNF-) α, interleukin (IL-) 1β, IL-6 and IL-10 are exemplary cytokines associated with ROS production [6] and are also hallmarks in different stages of COVID-19 [3,7]. Oxidative stress has been identified as a pathway towards neurodegenerative [8] and chronic kidney disease [9], as well as acute lung injury [10]. As a consequence, antioxidative co-treatment in critically ill patients has been frequently discussed and investigated for organ protective reasons [11], a consideration which may also apply for patients suffering from severe COVID-19 [12]. While the current evidence is sparse, the essential trace elements selenium (Se) and zinc (Zn) have hopeful prospects [13,14]. The Se-containing amino acid selenocysteine constitutes the active site of selenoproteins and provides oxidoreductase functions to selenoenzymes, essentially involved in the physiological immune response. Among the Se-dependent processes are macrophage signaling and cytotoxic activity of natural killer (NK) cells, as well as T cell differentiation and proliferation [15]. Zn is involved in regulating cellular pathways in innate and adaptive immunity [16] and plays a crucial role in lymphocyte maturation and development [17]. While these effects alone might strengthen host defense against viral pathogens, ribonucleic acid (RNA) viruses such as influenza A, hepatitis C and human immunodeficiency virus strongly rely on ROS production, which might even promote their viral replication and genome mutation rate [18]. Not surprisingly, these infectious diseases were associated with decreased Se levels and reduced glutathione peroxidase (GPx) activity, a major subgroup of the selenoprotein family with potent anti-oxidative and anti-inflammatory properties [19]. SARS-CoV-2 is another RNA virus, and Se as well as Zn deficiencies were detected in respective patients in recent German studies. Low blood levels served as valid predictors for the mortality risk, supporting the rationale to initiate Se and Zn supplementation trials in COVID-19 [20,21]. 

Here, we provide the first data on the feasibility and effects of Se and Zn supplementation in COVID-19 intensive care unit (ICU) patients with severe ARDS. To this end, we explored the potential relationships between different Se status biomarkers, Zn and relevant immunological, as well as clinical parameters.

## 2. Materials and Methods

### 2.1. Study Design and Patients

This is an observational single-center study at the University Hospital Wuerzburg, which adheres to the STROBE-Guidelines [22]. The institutional review board of the University of Wuerzburg waived the need for ethic approval (63/20-kr, 25 March 2020 and 20200528 01, 5 June 2020) due to sole retrospective chart review in conjunction with routine clinical and laboratory diagnostics. Informed consent was not necessary according to local legislation (Bayerisches Krankenhausgesetz, Art. 24, Abs. 4). The study period (20 March to 31 October 2020) was chosen to cover the first wave of COVID-19 in Germany and ICU patients with a confirmed SARS-CoV-2 infection [23] were consecutively screened for eligibility. Patients with moderate to severe ARDS, who were allocated to the ARDS and extracorporeal membrane oxygenation (ECMO) center of the University Hospital Wuerzburg, were further considered for the study. To be finally included, the individual chart review had to provide information about Se and Zn levels on admission and/or after 10 to 14 days of intensive care. Assignment to any other ICU of the University Hospital Wuerzburg and missing nutrient status were the two exclusion criteria. The allocation of patients was performed by an interdisciplinary committee of clinical experts.

### 2.2. Micronutrient Supplementation and Nutrition

Micronutrient supplementation was based on the local COVID-19 ICU standard operating procedures at the ARDS and ECMO center of the University Hospital Wuerzburg. It was started on the first day of intensive care and continued until discharge or death. Patients received intravenous administration of 1.0 mg Se as selenite (Biosyn Arzneimittel GmbH, Fellbach, Germany) daily and different combinations of artificial nutrition. Fresubin^®^ HP Energy (*n* = 21), Fresubin^®^ Renal (*n* = 9), Diben^®^ (*n* = 12), Survimed^®^ OPD (*n* = 4; each Fresenius Kabi Austria GmbH, Graz, Austria) and Cernevit^®^ + ADDEL TRACE^®^ (*n* = 22; Baxter Deutschland GmbH, Unterschleißheim, Germany) provided various amounts of Zn and Se, among other micronutrients. SmofKabiven^®^ (*n* = 22; Fresenius Kabi Austria GmbH, Graz, Austria) contained Zn. Monitoring of the nutrient status included a full assessment of Se, selenoprotein P (SELENOP), GPx3 and Zn on admission as well as after 10 to 14 days of intensive care. Serum samples were prepared for transport at −80 °C and analyzed in the Institute of Experimental Endocrinology (Charité-Universitätsmedizin Berlin, Berlin, Germany) as described recently [20,21]. Concentrations of Se and Zn were determined using total reflection X-ray fluorescence (S4 T-STAR, Bruker Nano GmbH, Berlin, Germany). SELENOP was quantified with a commercial ELISA-kit (selenOtest ELISA, selenOmed GmbH, Berlin, Germany) according to manufacturer’s instructions. The activity of GPx3 was assessed via consumption of nicotinamide adenine dinucleotide phosphate (NADPH) at 340 nm in a coupled enzymatic assay as initially described by Flohé and Günzler [24]. To this end, samples were incubated with reduced glutathione, NADPH, sodium acide (NaN3) and glutathione reductase. The enzymatic reaction was started with hydrogen peroxide. Reference ranges were adopted from the European Prospective Investigation into Cancer and Nutrition (EPIC) study, where similar methods have been used on a large number of samples in healthy adults [25,26].

### 2.3. Data Collection

Clinical data were extracted from a patient data management system (COPRA6 RM1.0, COPRA System GmbH, Berlin, Germany). ARDS was classified according to the Berlin definition [27]. Standard parameters were collected at the laboratory of the University Hospital Wuerzburg, including C reactive protein (CRP), procalcitonin (PCT), IL-6, total lymphocyte count and immunoglobulin G (IgG). IL-1β, IL-8, IL-10, IL-12, TNF-α, and CXCL-10 were routinely analyzed by an external diagnostics provider (Ganzimmun Diagnostics AG, Mainz, Germany) from deep-frozen serum samples (−80 °C) using the BD CBA Human Inflammatory Cytokines Kit (BD Biosciences, San Jose, CA, USA) according to instruction. Fluorescence-activated cell sorting was conducted at a Navios cytometer (Beckman Coulter, Krefeld, Germany) at the University Hospital Wuerzburg. A minimum of 3000 events per lymphocyte gate were recorded and the following anti-human antibodies were used: anti-CD45-Krome-Orange, anti-CD14-APCA700, anti-CD3-FITC, anti-CD4-APC, anti-CD8-ECD, anti-CD56/CD16-APC A750, anti-CD19-PC7, anti-CD38-PC5.5, anti-CD27-ECD, anti-CD20-APC750 (each Beckman Coulter, Krefeld, Germany) and anti-IgD-FITC (BD Biosciences, San Jose, CA, USA). Reference values are based on the literature [28,29].

The primary explorative endpoint was to evaluate the kinetics of trace elements under supplementation during routine intensive care. As secondary endpoints, we evaluated the inflammatory immune response and PaO_2_/FiO_2_ ratio, as a marker of ARDS severity. Further exploratory outcomes included the length of ICU stay and mechanical ventilation, sequential organ failure assessment (SOFA) score, ECMO, nosocomial infections and mortality. PaO_2_/FiO_2_ ratio and SOFA score were calculated daily. For correlation analyses, the respective scores on the day of blood sampling for micronutrient assessment were used. Our study covers the whole course of intensive care. Therefore, the term “non-survivor” refers to ICU mortality and “survivor” is defined as survival upon ICU discharge.

### 2.4. Statistical Analysis

Due to small sample sizes, the normality of the data could not be assumed. Categorial variables are presented as absolute numbers and percentages, while continuous variables are expressed as the median ± interquartile range (IQR, 25–75%). Wilcoxon’s paired test was used to assess longitudinal changes. The Mann–Whitney rank-sum test was applied to compare numeric variables and Fisher’s exact test was used for categorial data. Associations between different variables were correlated according to Spearman. Linear regression was applied to the graphs in Figure 3, and the 95% confidence interval is also shown. Statistical significance was considered as *p* < 0.05. Data were analyzed with Microsoft Office^®^ 365 ProPlus (Microsoft™, Redmond, WA, USA) and GraphPad Prism^®^ Version 9.0.2 (GraphPad Software™, San Diego, CA, USA).

## 3. Results

### 3.1. Demographics and Baseline Characteristics

**Figure 1:**
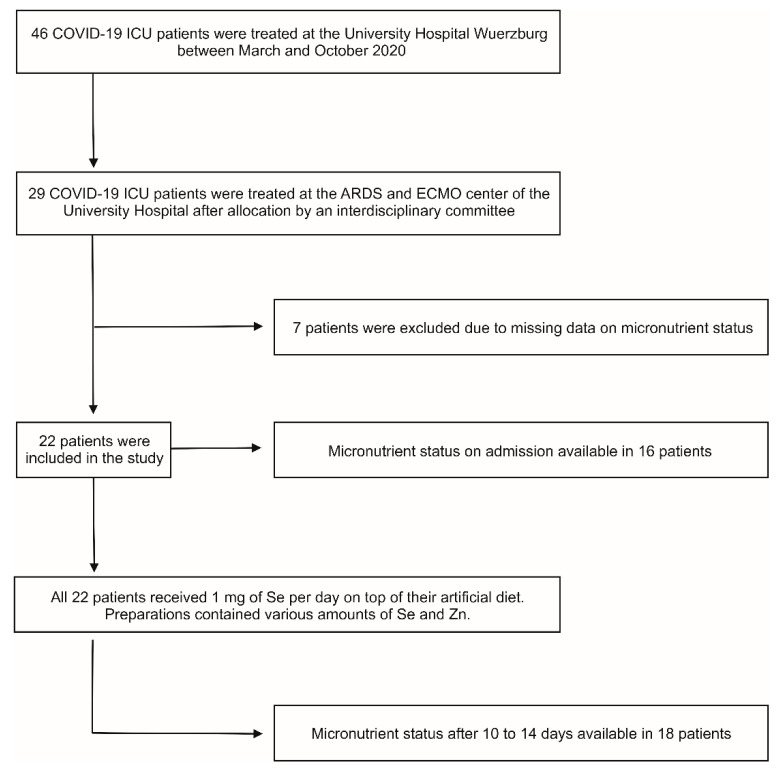
Flow diagram of retrospective study inclusion. All 22 patients received high-dose selenium (Se) as selenite and artificial diet, which additionally included various amounts of zinc (Zn). ARDS, acute respiratory distress syndrome; ECMO, extracorporeal membrane oxygenation; ICU, intensive care unit.

In total, 22 patients were included in this study between March and October 2020 (Figure 1). Overall, 64% were male, 36% were female, and the median age was 60.5 years (50–69). On admission to ICU, a median SOFA score of 15 (13–16) indicated high severity of illness. In addition, 95% of the patients suffered from severe ARDS at any time during intensive care and 64% survived upon discharge from ICU (Table 1).

### 3.2. Temporal Development of Inflammation, Immune Cell Numbers and Micronutrients during Supplementation

Patients had significantly elevated levels of CRP, PCT and IL-6 on admission, when compared to normal reference values. Alongside this, the median levels of IL-10, IL-12 and CXCL-10 were also increased. Lymphocyte counts were depleted, with CD8^+^ T cells and NK cells being affected the most. However, over the course of the ICU stay, inflammatory parameters normalized and cell counts were restored (Table 2).

**Figure 2:**
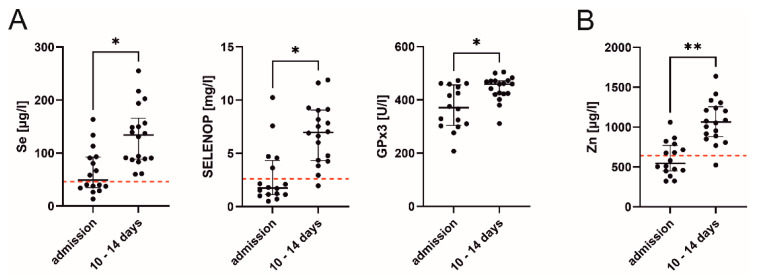
Supplementation increased the (**A**) selenium (Se) status biomarkers and (**B**) zinc (Zn) levels over the course of intensive care. Each black dot represents an individual patient. The lower end of each reference range is indicated by a red dashed line. Ranges were adopted from the European Prospective Investigation into Cancer and Nutrition (EPIC) study. However, glutathione peroxidase 3 (GPx3) levels have not been determined in a large patient collective so far. Therefore, a validated and comparable reference range is not available. SELENOP, selenoprotein P. *p* < 0.05 (*), *p* < 0.01 (**).

On admission to ICU, 50% of patients with available micronutrient status (*n* = 8) demonstrated a substantial Se deficiency. SELENOP levels were also reduced in 69% of patients (*n* = 11). After 10 to 14 days of supplementation, Se levels significantly increased to the normal range in all patients (*p* = 0.027), as well as SELENOP (*p* = 0.016), with only one case remaining below reference range. Accordingly, a significant increase in GPx3 activity over time (*p* = 0.021) was observed. Low levels of Zn were initially observed in 56% of patients (*n* = 9), and again normalized within two weeks of supplementation (*p* = 0.002) (Figure 2).

**Figure 3:**
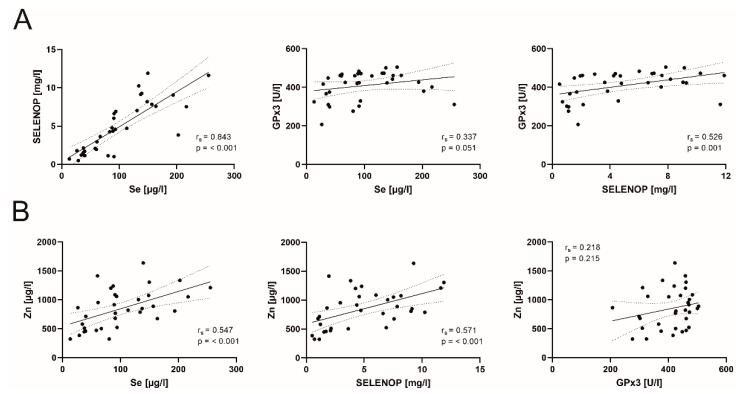
(**A**) Correlation analyses of selenium (Se), selenoprotein P (SELENOP) and glutathione peroxidase 3 (GPx3). (**B**) Correlation of Se status biomarkers and zinc (Zn). Spearman’s correlation coefficients (r_s_) and respective *p*-values are indicated. Linear regression was applied and the 95% confidence interval is shown.

There were significant associations between micronutrients in our patients (Figure 3). We found correlations between Se and SELENOP (r_s_ = 0.843, *p* = < 0.001), Se and Zn (r_s_ = 0.547, *p* = < 0.001), SELENOP and GPx3 (r_s_ = 0.526, *p* = 0.001) and SELENOP and Zn (r_s_ = 0.571, *p* = < 0.001).

### 3.3. Potential Clinical Relevance of Supplemental Micronutrients for Critically Ill COVID-19 Patients

**Figure 4:**
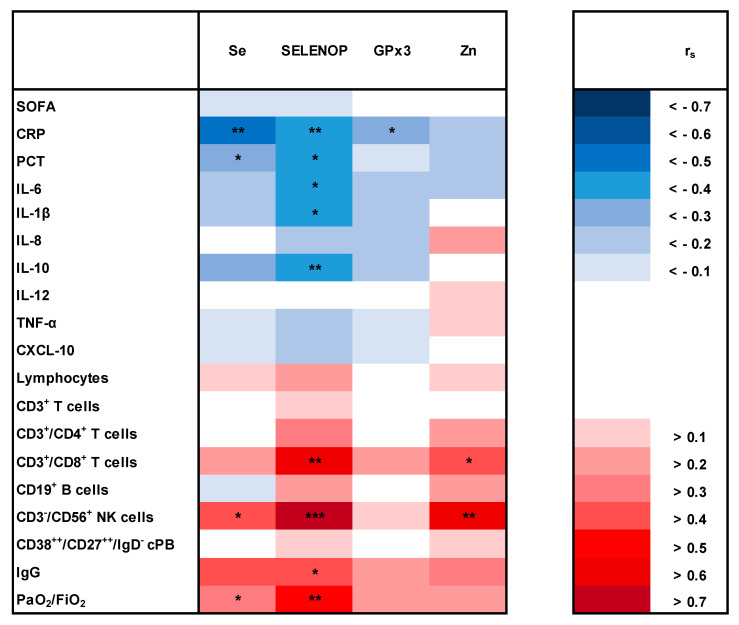
Correlation matrix of nutritional status and inflammation. Red colors indicate a positive and blue colors a negative correlation coefficient (r_s_). *p* < 0.05 (*), *p* < 0.01 (**), *p* < 0.001 (***). CPB, circulating plasmablasts; CRP, C-reactive protein; Ig, immunoglobulin; IL, interleukin; GPx3, glutathione peroxidase 3; NK, natural killer; PCT, procalcitonin; Se, selenium; SELENOP, selenoprotein P; SOFA, sequential organ failure assessment score; TNF, tumor necrosis factor; Zn, zinc.

Sufficient Se homeostasis was furthermore associated with reduced parameters of inflammation and restored numbers of lymphocytes (Figure 4). Se was inversely correlated with CRP (r_s_ = −0.482, *p* = 0.005) and PCT (r_s_ = −0.371, *p* = 0.034) and positively associated with the number of NK cells (r_s_ = 0.452, *p* = 0.045). In addition, SELENOP was negatively correlated with CRP (r_s_ = −0.495, *p* = 0.003), PCT (r_s_ = −0.413, *p* = 0.017), IL-6 (r_s_ = −0.429, *p* = 0.013), IL-1β (r_s_ = −0.440, *p* = 0.012) and IL-10 (r_s_ = −0.461, *p* = 0.008). SELENOP was furthermore associated with higher numbers of CD8^+^ T cells (r_s_ = 0.636, *p* = 0.003), NK cells (r_s_ = 0.772, *p* = < 0.001) and total IgG (r_s_ = 0.493, *p* = 0.027). Alongside reduced inflammation, PaO_2_/FiO_2_ ratios were improved as a function of Se (r_s_ = 0.356, *p* = 0.042) and SELENOP (r_s_ = 0.504, *p* = 0.003). Further exploratory endpoints such as SOFA score, time on mechanical ventilation and in the ICU, ECMO and nosocomial infections were not related to Se biomarkers.

**Figure 5:**
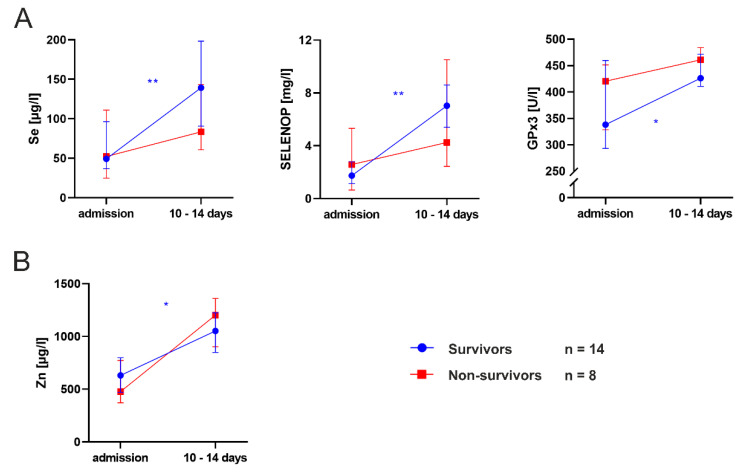
Effects of trace element supplementation on (**A**) selenium (Se) and (**B**) zinc (Zn) concentrations in survivors and non-survivors. Wilcoxon’s paired test was used to compare micronutrient levels between admission and day 10 to 14. *p* < 0.05 (*), *p* < 0.01 (**). GPx3, glutathione peroxidase 3; SELENOP, selenoprotein P.

In comparison to patients with a fatal outcome (*n* = 8), survivors (*n* = 14) significantly responded to supplementation with an increase in Se (*p* = 0.008), SELENOP (*p* = 0.004), GPx3 (*p* = 0.039) and Zn levels (*p* = 0.020) over the course of the ICU stay (Figure 5). Decedents had a median ICU course of 17.5 days (12–22), whereas patients with a favorable outcome were treated for significantly longer (40 days, 20–44; *p* = 0.025). Despite a similar supplementation regimen, survivors tended to have higher Se levels after 10 to 14 days of intensive care compared to non-survivors (*p* = 0.075). However, this finding might be biased by small n-numbers.

## 4. Discussion

The present pilot study highlights a potential benefit of micronutrient supply in a highly selective study population of critically ill patients with severe COVID-19 induced ARDS, requiring ECMO in almost 70% of the cases. On admission, Se biomarkers and Zn concentrations were below the reference range. We observed excessive inflammatory parameters and reduced lymphocyte counts. Intravenous Se supplementation in the form of selenite (1.0 mg per day) in addition to fortified artificial nutrition containing Se and Zn was feasible and effective in restoring an adequate micronutrient status within two weeks of intensive care. This was associated with reduced inflammation, increasing lymphocyte counts and clinical recovery. In total, 64% of the patients survived intensive care, which compares favorably with previously published mortality rates [30,31,32,33].

The antioxidative redox system relies on selenoproteins such as SELENOP and GPx3 to catalyze the neutralization of reactive oxygen and nitrogen species. In addition to this direct, antioxidative effect, the transporter SELENOP facilitates the distribution of Se to target tissues, where it directly affects the expression and enzymatic activity of further protective selenoproteins. Either a massive accumulation of ROS or a shortage in Se supply can tip the redox equilibrium towards oxidative stress. Severe COVID-19 is characterized by systemic inflammation [34], which is a predestined condition, where arising oxidative stress can drive multi organ damage [35]. Low levels of Se have been associated with higher morbidity and mortality in critically ill patients [36,37], and Se supplementation improved inflammation and pulmonary mechanics in ARDS [38]. A trial including 249 participants found reduced mortality rates in septic patients receiving high-dose Se compared to placebo [39], whereas the randomized REDOXS study in 1223 critically ill patients did not reveal any clinical benefits of Se and antioxidant supplementation [40]. As additional trials are rather small and heterogenous with high risk of bias [41] and following meta-analyses underlined the resulting ambiguous findings [42,43], international guidelines currently do not recommend the general use of antioxidants in critically ill patients but consider supplementation in cases with proven deficiency [44]. However, respective data in the context of severe COVID-19 induced ARDS are not yet available and it is unclear whether previous results can be easily translated into the ongoing pandemic.

At the ARDS and ECMO center of the University Hospital Wuerzburg, standard operating procedures during the first wave of COVID-19 included a routine micronutrient assessment on admission and after 10 to 14 days of intensive care. The analyses of the serum samples required an external laboratory, which involved a transportation route of 500 km, consolidated shipment for logistical reasons and a consequential delay in the reporting of the results. The local ICU protocol therefore inevitably implemented preliminary nutrient supplementation, even with pending evidence of a deficiency. Retrospectively, we found Se and SELENOP levels to be below the reference range in 50% and 69% of our patients on admission to ICU, respectively. Our supplementation strategy was then able to effectively remedy these deficiencies within two weeks of intensive care. Alongside, GPx3 activity markedly increased, indicating restored systemic and cell-specific protection against oxidative stress via the reduction of hydrogen peroxide at the expense of glutathione [45]. The three complementary biomarkers, Se, SELENOP and GPx3, showed the expected correlations, suggesting that sodium selenite infusions are an easy, efficient and straightforward way to improve antioxidant defense mechanisms.

This might also apply for Zn, another essential trace element with multi-layered impacts on the immune system, ROS production and degradation [46]. Its indispensable role, especially during infection, is broadly acknowledged [16,17,47], providing a rationale for supplementation in COVID-19 patients [14]. While Zn was below the reference range in 56% of the patients on admission, adjuvant and Zn-containing artificial nutrition could normalize levels in the majority of patients up to day 10 to 14. Again, Zn was closely associated with Se and SELENOP as described before [21]. Se biomarkers were correlated with reduced parameters of inflammation, especially CRP, which has been identified as a reliable predictor of disease severity and outcome in COVID-19 [48,49]. Contrary to former beliefs, recent studies questioned the role of an excessive cytokine storm in severe COVID-19 [50] and rather suggest a balanced elevation of pro- as well as anti-inflammatory cytokines. Accordingly, IL-6 and IL-10 levels were both inversely associated with SELENOP in our study. This is partially in contrast to an animal model of Se deficiency-induced renal inflammation, where disruption of selenoproteins led to an initiation of the NF-κB pathway with increased IL-6 and reduced IL-10 concentrations [51]. On the other hand, IL-6 can directly suppress hepatic biosynthesis of SELENOP [52], which might result in declining Se levels during systemic inflammation, independent of the Se baseline.

IL-6 production can be strongly amplified by IL-1β, which is another signature cytokine of monocytes and macrophages during innate immune response [53]. Cytokine release, as well as phagocytic and migratory properties of macrophages, can be modulated by Se levels and selenoproteins [15], this being one possible explanation for the interrelations observed in this study. Innate antiviral defense also relies on cytotoxic capacities of NK and CD8^+^ T cells. Both lymphocyte subsets showed a distinct reduction in numbers on admission to ICU, which is in line with studies pointing to functional exhaustion and an impaired cytotoxic response in COVID-19 patients [54]. Excessive production of IL-6 might suppress lymphopoiesis [55] and SARS-CoV-2 can induce cell death via Fas/FasL-dependent signaling [56]. We have previously shown that inflammation and viral loads were drastically reduced after two weeks of intensive care in patients with COVID-19 induced ARDS and antibody titers against the spike receptor binding domain of the virus were fully established at this point of time [57,58]. On the one hand, both mechanisms could explain the initial lymphocytopenia and recovery of the lymphocyte subsets regardless of the micronutrient status. On the other hand, trace element supplementation might well play a supportive role, as increasing levels of Se, SELENOP, and Zn were strongly associated with the restoration of NK and CD8^+^ T cell subsets in our patients. These findings complement previous observations, where Se and Zn supplementation has shown beneficial effects on NK and T cell numbers as well as cytotoxic capacity [59]. Our data further suggest a relationship between SELENOP and total IgG levels, which may serve as a surrogate parameter for B cell function.

We speculate that an improvement in cellular and humoral immune response might promote virus clearance and, in combination with reduced systemic inflammation, support the resolving of severe ARDS [60]. The positive correlation between Se status and PaO_2_/FiO_2_ ratios, as well as a trend towards higher Se levels in survivors compared to non-survivors, might underline this assumption. Patients with a favorable outcome even seemed to respond better to supplementation with a more pronounced increase, especially in Se and SELENOP. Rising kinetics of Se biomarkers and Zn have been used as a prognostic tool before, and COVID-19 survival was accompanied by higher nutrient levels compared to fatal cases [20,21]. However, it has to be noted that our study does not provide additional insights into potential relationships between Se biomarkers, Zn and further clinical outcomes, such as SOFA score, duration of mechanical ventilation, length of ICU stay, ECMO treatment and nosocomial infections. This is in line with previous studies on Se supplementation in cardiac surgery [61,62] and septic shock [63], where no persistent effect on the SOFA score, duration of mechanical ventilation and length of ICU stay was found. All of these multifactorial parameters and outcomes mirror the complexity of intensive care far beyond nutritional support and are immensely influenced by complications such as bleeding, thromboembolic events, hemodynamic instability or other conditions.

In this light, the retrospective design and the small number of patients, especially when divided into survivors and non-survivors, are the major limitations of our explorative study. Datasets for micronutrients and immunologic parameters are not complete in all cases and limit the possibility to include additional patients. However, to the best of our knowledge, this is the first study to investigate the feasibility and potential clinical relevance of micronutrient supplementation in patients with severe COVID-19 ARDS. Even though this study reflects real world data from clinical practice, the observational results remain correlative and do not necessarily show a causative relationship. Allocation, treatment, nutrition and sampling were solely at the hands of the responsible ICU-team and not affected by this study. Therefore, the risk of selection bias cannot be excluded and a control group without micronutrient supplementation is not available. Individual combinations of artificial diets may have moreover led to slightly different amounts of supplemented micronutrients during the course of intensive care. Most patients were referred to our tertiary care center via the German ARDS network, whereby information about early stages of the disease cannot be provided. Although daily Se intake of 1.0 mg is well below the toxic dose [64], our study is not sufficiently powered to evaluate safety and side effects of the supplementation.

## 5. Conclusions

Taken together, the present findings strengthen the notion on a clinical significance of adequate Se and Zn supply for critically ill patients with severe COVID-19 ARDS. Commonly observed deficiencies can be effectively compensated by applying the outlined supplementation strategy. Se and Zn might be involved in the reduction in inflammation and the restoration of critical lymphocyte counts for the cytotoxic immune response, which may further translate into clinical improvement. However, the results need to be considered within the limits of an observational study, so that adequately designed trials are encouraged to fully elucidate the clinical relevance of micronutrient supplementation in patients with severe COVID-19.

## Figures and Tables

**Table 1:** Demographics and course of intensive care.

	All Patients *n* = 22	Survivors * n = 14	Non-Survivors *n* = 8	*p*
Female, No. patients (%)	8 (36)	4 (29)	4 (50)	0.386
Male, No. patients (%)	14 (64)	10 (71)	4 (50)	0.386
Age, years (median, IQR)	60.5 (50–69)	53 (48–68)	66 (63–68)	0.113
Charlson comorbidity index (median, IQR)	2 (2–4)	2 (2–3)	3.5 (2–5)	0.079
SOFA score, admission (median, IQR)	15 (13–16)	15 (13–16)	16 (15–16)	0.405
Minimal PaO_2_/FiO_2_, mmHg (median, IQR)	60 (51–69)	65 (58–71)	56 (51–62)	0.192
Severe ARDS, No. patients (%)	21 (95)	13 (93)	8 (100)	0.999
VvECMO, No. patients (%)	15 (68)	9 (64)	6 (75)	0.999
Renal replacement therapy, No. patients (%)	17 (77)	9 (64)	8 (100)	0.115
Intravenous corticosteroid therapy, No. patients (%)	14 (64)	8 (57)	6 (75)	0.649
Duration of intensive care, days (median, IQR)	24.5 (15–42)	40 (20–44)	17.5 (12–22)	**0.025**

* Survival upon discharge from the intensive care unit. ARDS, acute respiratory distress syndrome; IQR, interquartile range; No., number of; SOFA, sequential organ failure assessment; VvECMO, veno-venous extracorporeal membrane oxygenation.

**Table 2:** Immune response.

	Reference Range	Admission (*n* = 22)	10–14 Days (*n* = 19)	*p*
CRP, mg/dL	0–0.5	24 (19–32)	15 (8–21)	**0.049**
PCT, ng/mL	0–0.5	1.4 (0.6–4.5)	1.5 (0.6–3.1)	0.671
IL-6, pg/mL	0–7	501 (168–1211)	110 (54–306)	**<0.001**
IL-1β, pg/mL	0–4.9	2.3 (1.8–3.2)	1.9 (1.3–3.1)	0.297
IL-8, pg/mL	0–1648	199 (87–407)	162 (111–428)	0.359
IL-10, pg/mL	0–1.8	16.6 (8.4–25.4)	15.6 (7.6–22.8)	0.652
IL-12, pg/mL	0–0.6	1.7 (1–3.5)	1.8 (1.3–2.2)	0.672
TNF-α, pg/mL	0–2.9	0.8 (0.1–1.9)	0.6 (0.1–1.2)	0.750
CXCL-10, pg/mL	0–80	759 (278–874)	583 (353–679)	0.734
Lymphocytes, × 1000/µL	1–4	0.9 (0.7–1.2)	1.4 (0.9–1.7)	0.188
CD3^+^ T cells, × 1000/µL	718–2494	804 (236–1100)	1259 (910–1420)	**0.039**
CD3^+^/CD4^+^ T cells, × 1000/µL	456–1492	609 (188–741)	585 (558–772)	0.250
CD3^+^/CD8^+^ T cells, × 1000/µL	272–1144	134 (61–317)	382 (324–745)	**0.008**
CD19^+^ B cells, × 1000/µL	112–622	107 (78–169)	140 (97–197)	0.547
CD3^−^/CD56^+^ NK cells, × 1000/µL	82–760	56 (21–100)	157 (127–213)	0.195
CD38^++^/CD27^++^/IgD^−^ cPB, × 1000/µL	1–3	3.9 (0.5–10.9)	4.9 (3.3–12.2)	0.945
IgG, mg/dL	690–1600	785 (600–907)	1086 (817–1197)	0.193

The table shows median values and interquartile ranges. Datasets are not complete in respect to immune cell counts and interleukin levels for all patients. CPB, circulating plasmablasts; CRP, C-reactive protein; Ig, immunoglobulin; IL, interleukin; NK, natural killer; PCT, procalcitonin; TNF, tumor necrosis factor.

## Data Availability

The data presented in this study are available on request from the corresponding author. The data are not publicly available due to privacy regulations.
